# Utilization of a Gas-Sensing System to Discriminate Smell and to Monitor Fermentation during the Manufacture of Oolong Tea Leaves

**DOI:** 10.3390/mi12010093

**Published:** 2021-01-17

**Authors:** Ting-Shiang Tseng, Mei-Hui Hsiao, Po-An Chen, Shu-Yen Lin, Shih-Wen Chiu, Da-Jeng Yao

**Affiliations:** 1 Institute of NanoEngineering and MicroSystems, National Tsing Hua University, Hsinchu 30013, Taiwan; shanksgoto65@gapp.nthu.edu.tw (T.-S.T.); mhhsiao@gapp.nthu.edu.tw (M.-H.H.); 2 Plant Technology Laboratories, Agricultural Technology Research Institute, Hsinchu 30093, Taiwan; chenpoan@mail.atri.org.tw; 3 Department of Horticulture and Landscape Architecture, National Taiwan University, Taipei 10617, Taiwan; sylin@ntu.edu.tw; 4 Enosim Bio-Tech Co., Ltd., Hsinchu 30013, Taiwan; scott_chiu@enosim.com.tw

**Keywords:** MOS gas sensor, E-nose, LDA, on-line monitoring

## Abstract

The operational duration of shaking tea leaves is a critical factor in the manufacture of oolong tea; this duration influences the formation of its flavor and fragrance. The current method to control the duration of fermentation relies on the olfactory sense of tea masters; they monitor the entire process through their olfactory sense, and their experience decides the duration of shaking and setting. Because of this human factor and olfactory fatigue, it is difficult to define an optimum duration of shaking and setting; an inappropriate duration of shaking and setting deteriorates the quality of the tea. In this study, we used metal-oxide-semiconductor gas sensors to establish an electronic nose (E-nose) system and tested its feasibility. This research was divided into two experiments: distinguishing samples at various stages and an on-line experiment. The samples of tea leaves at various stages exhibited large differences in the level of grassy smell. From the experience of practitioners and from previous research, the samples could be categorized into three groups: before the first shaking (BS1), before the shaking group, and after the shaking group. We input the experimental results into a linear discriminant analysis to decrease the dimensions and to classify the samples into various groups. The results show that the smell can also be categorized into three groups. After distinguishing the samples with large differences, we conducted an on-line experiment in a tea factory and tried to monitor the smell variation during the manufacturing process. The results from the E-nose were similar to those of the sense of practitioners, which means that an E-nose has the possibility to replace the sensory function of practitioners in the future.

## 1. Introduction

**Figure 1:**
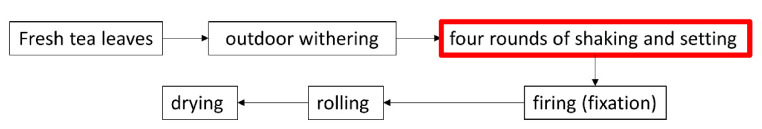
Manufacture of tea leaves.

Oolong tea is a typical kind of Taiwanese tea famous for its enriched flavor and elegant fragrance. Teas are categorized into three major groups depending on the level of fermentation: (a) unfermented green tea, (b) partially fermented oolong tea, and (c) fully fermented black tea. Taiwan is the main manufacturer and global consumer of oolong tea. The manufacturing process of oolong tea includes withering, four rounds of shaking and setting (for fermentation or oxidation), firing (fixation), and rolling and drying as shown in Figure 1. Among all stages of the process, fermentation is the most crucial, because it has a great influence on the tea’s flavor and aroma [1]. Oxidation occurs during fermentation [2,3], forming the chemical components that are primarily responsible for deciding the quality of the finished tea [4,5]. This paper establishes a novel gas-sensing system to distinguish tea samples at various stages and reports an on-line experiment during manufacturing to monitor the volatile organic compounds.

During fermentation, the tea leaves typically undergo shaking and setting in four cycles. In general, each cycle endures for approximately two hours and depends on the temperature, moisture, and content of the leaves. Some chemical variation occurs with the volatile organic compounds (VOCs) of tea leaves after cycles of shaking and setting. Tea practitioners need to smell the tea aroma personally to determine the duration of shaking and setting. The first two shakings are mild, leading to the transport of water from the stalk to the leaves; water is emitted through the stoma [6,7]. The force of the latter two shakings intensifies, enhancing the oxidation [8]. The grassy smell of tea leaves transforms into a floral aroma after the shaking and setting. The duration of shaking and setting is determined by olfactory senses and experience. Practitioners constantly monitor the smell and evaluate the aroma to decide when to shake or set. An incorrect duration of shaking and setting leads to an over-fermented or under-fermented product; both situations not only generate a strange smell but also diminish the quality of the tea.

To optimize the flavor and quality of tea, practitioners play an important role during manufacture. The smell of tea alters during the setting. Some volatile organic compounds with a grassy odor dominate the fermentation; practitioners monitor the variation of this grassy odor and control the duration of shaking and setting to derive the status of the tea at an optimized point. Grassy odors increase after each shaking and then decrease with time during setting. When the smell diminishes to a degree of balance, that condition becomes the optimum duration of shaking. On the contrary, the compounds with a floral odor increase during setting. The shaking and setting not only decrease the grassy-disliked odor but also increase the floral-targeted flavor [9]. The tea sample can be classified into two groups (before shaking groups and after shaking groups) according to the smell property. Except for the two groups, the sample before first shaking (BS1) is easily influenced by the weather, humidity, and daylight on the day the tea leaves are picked; its smell differs from that of other groups of samples before shaking. In sum, tea leaves at various stages can be divided into three groups: before first shaking (BS1), before shaking group, and after shaking group.

An electronic nose (E-nose) has had several successful applications in the food and beverage industry [10]. In this research, we conducted two experiments to ensure that an E-nose system can be applied in tea factories to monitor the smell variation of fermentation. First, we sampled tea leaves at various stages during manufacture-before the first shaking (BS1), after the first shaking (AS1), before the second shaking (BS2), before the third shaking (BS3), after the third shaking (AS3), before the fourth shaking (BS4), and after the fourth shaking (AS4)—in total seven samples. These samples exhibited large differences in the level of grassy smell, which an E-nose must distinguish so that we can evaluate the feasibility of its application to monitor on-line. After verifying the functionality of an E-nose, we proceeded to a second experiment in which an E-nose was set-up in a tea factory to monitor the smell variation and to ensure the possibility of replacing the olfactory sense of practitioners in the future.

## 2. Distinguishing the Samples at Various Stages

### 2.1. Sensor Selection

The most important role of an E-nose arises from the array of sensors. In this research, we applied metal-oxide-semiconductor (MOS) gas sensors (Figaro USA, Inc., Arlington Heights, IL, USA and Nissha FIS, Inc., Osaka, Japan) in our E-nose system; a similar setup was used to classify the aroma of black tea in previous research [11]. The MOS gas sensors utilize oxidation-reduction as a sensing mechanism. Several studies have worked on the improvement for selectivity [12,13,14] and electrical property [15,16] of MOS gas sensors. The reliability and limitation issues have also been solved by application of composite material [17,18,19]. The resistance of a sensor varies with both the nature of the surrounding gases and their concentration [20], which can be evaluated by measuring the voltage of the sensors. The MOS gas sensors have three advantages for monitoring the smell of tea [21]: first, the sensing mechanism is reduction-oxidation, which is beneficial for repeatable use; second, the rapid response and recovery interval can immediately inform the actual status of a smell [22,23]; third, the sensor array is easily integrated with a measurement circuit. The target gases of the sensors vary from alcohols to volatile organic compounds of several kinds as shown in Table 1. Each sensor has a distinct chemical property, which leads to a distinct target gas. Using various sensors in an E-nose, we can analyze the affinity of various compounds among those sensors.

Besides a sensor array, the E-nose system comprises a measurement circuit and a data acquisition (DAQ) system. After the sensor senses the sample gas, the electric signals are transmitted to a DAQ system through an electric circuit; the DAQ system then sends the data to a computer. The sensor array, DAQ system, and gas route were developed by Enosim Bio-Tech Co., Ltd. (Hsinchu, Taiwan).

### 2.2. Gas-Sensing System

**Figure 2:**
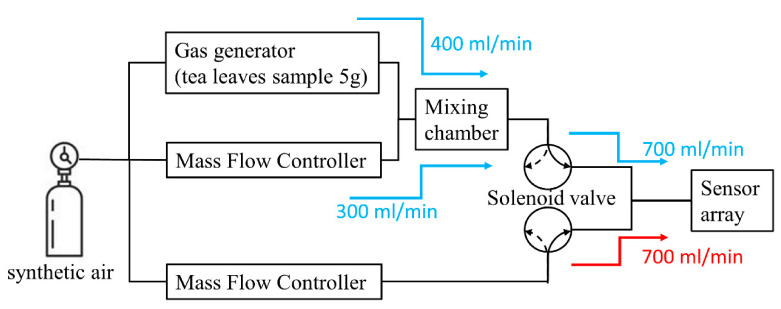
Gas-sensing system.

To set-up the gas system, we used a gas generator (Molecular Analysis LLC., Wilmington, DE, USA), a solenoid valve, two mass-flow controllers (Kofloc Corp., Kyoto, Japan), and a mixing chamber to establish a gas-measurement system as shown in Figure 2. During the experiment, there were two main steps for the gas-sensor system—adsorption and desorption. For adsorption (blue arrays in Figure 2), tea leaves samples were placed inside a gas generator. A carrier gas (synthetic air) was passed towards the gas generator that generated a gas from the sample tea leaves. Synthetic air was then mixed with the sample gas generated from the tea leaves in a mixing chamber for complete blending. After, the blended gas passed through the sensor arrays so that the sensors could detect the VOCs of the tea leaves. After 25 min, the sensors achieved a saturation level; we then desorbed the VOCs from the surface of the sensors. During desorption (red arrays in Figure 2), synthetic air was passed through the surface of the sensors to desorb the VOCs. Each experiment included five cycles of adsorption and desorption so that we derived five values of signals from a single experiment.

### 2.3. Introduction of Smell Variation

**Figure 3:**
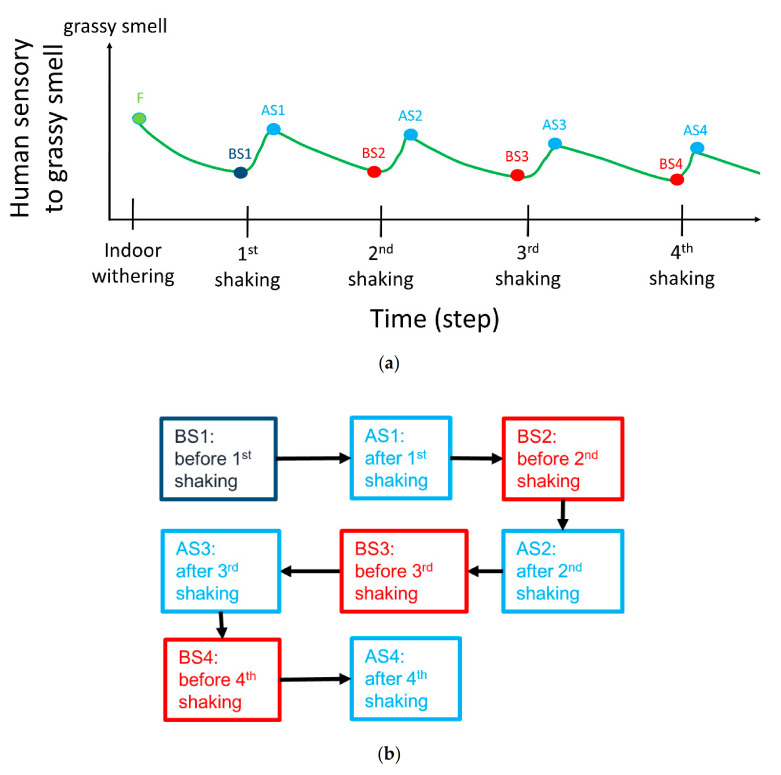
(**a**) Smell relation with tea manufacturing. (**b**) Classification of tea samples.

Tea practitioners typically take a grassy smell as the crucial factor for shaking because the smell variation of fermentation is dominated by that grassy smell. We hence took the level of the grassy smell as ordinate axis in Figure 3a, and the progress of the manufacturing process as the abscissal axis. To ensure the feasibility of an E-nose for samples with a large difference in the level of grassy smell, we sampled tea leaves at seven intervals before each shaking (BS*_n_*, *n* = 1, 2, and 4) and after shaking (AS*_n_*, *n* = 1, 2, 3, and 4). We selected these seven samples because tea practitioners and previous research [9] can classify these seven samples into three groups: before the first shaking (BS1), before shaking group (except BS1), and after shaking group as shown in Figure 3b. The sample for BS1, a purple block as shown in Figure 3b, presented an unsteady smell because the tea leaves were just picked from a farm, and their smell was easily influenced by the environment. The samples of the before-shaking group, red blocks as shown in Figure 3b, had a less grassy smell because the samples had experienced a duration of setting, which made the smell stable and diminished the grassy smell. The samples of the after-shaking group, red purple block shown in Figure 3b, had a grassier smell and required an interval for setting to decrease the grassy smell. In sum, the tea leaves at various stages were divided into three groups: before first shaking (BS1), before-shaking group (except BS1), and after-shaking group.

### 2.4. Results

**Figure 4:**
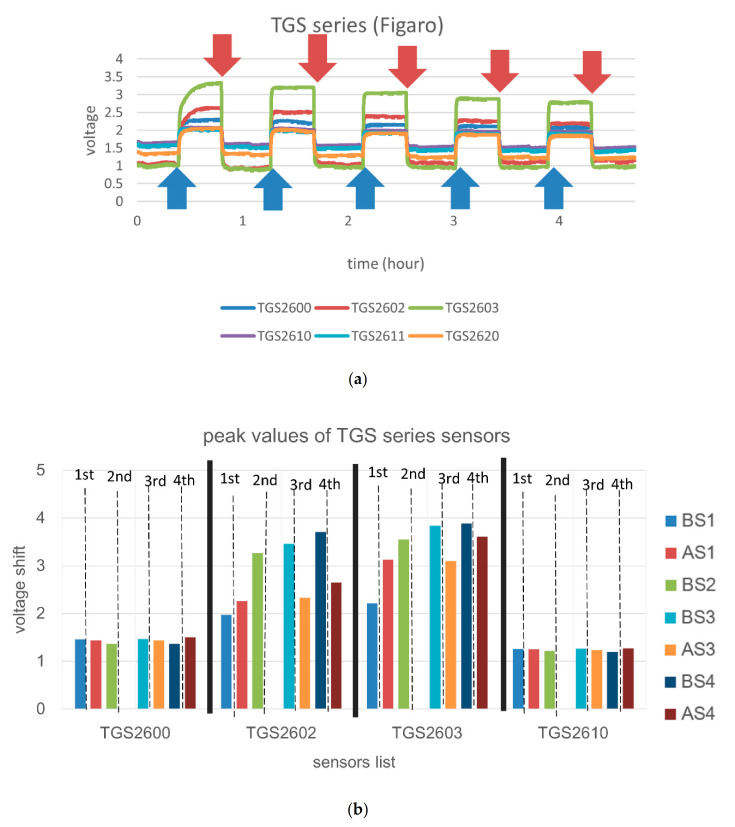

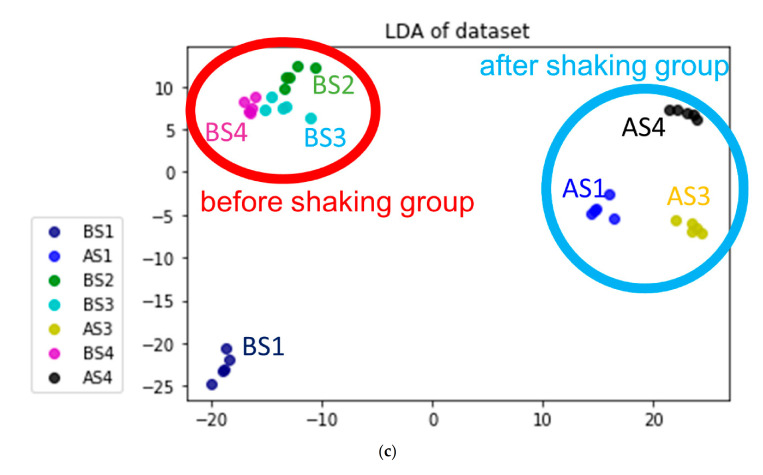
(**a**) Raw data of sensors with prefix ‘TGS-’; (**b**) statistical results of four TGS series sensors; (**c**) results of linear discriminant analysis (LDA).

Every experiment generated twelve sensor responses. First, we purged the sensors with synthetic air for a while to ensure the cleanliness of the sensor surface, so that the voltage values in Figure 4 remained constant at first. Second, we purged the gas from the tea leaf sample into the sensor chamber so that the sample gas molecules adsorbed on the sensor surface. The voltage increased with time until the decreased reaction achieved a saturated status; the voltage then remained constant for minutes. After that, we repeated four times the purging and adsorption to test the repeatability. Hence, each experiment consisted of five cycles of adsorption (blue arrays in Figure 4a) and desorption (red arrays in Figure 4a); we achieved five cycles of variation from the sensor responses. During the experiment, the variation of peaks decreased with time because the smell of the sample decreased gradually. We obtained peak values between desorption and adsorption from the measurement voltage data as shown in Figure 4b.

We applied linear discriminant analysis (LDA) to discriminate various samples of tea leaves; as shown in Figure 4c, the tea leaves before each shaking (BS2, BS3, and BS4) had a similar smell. The sample after each shaking (AS1, AS3, and AS4) also showed an identical smell. The smell of sample BS1 differed from others, coinciding with our expectation, as shown in Figure 3.

## 3. On-Line Experiment

### 3.1. Gas-Sensing System

Having verified the feasibility of the E-nose for samples with a large difference in the level of grassy smell, we proceeded to a tea factory and conducted on-line experiments to monitor using the E-nose the variations in smell during fermentation.

**Figure 5:**
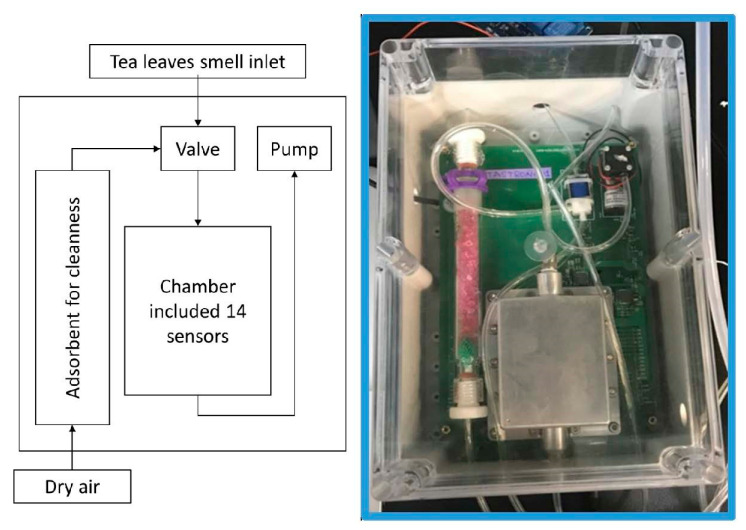
Gas-sensing system of on-line experiment.

The electronic nose consisted of (a) a sensor array, (b) a micro-pump with solenoid valves, (c) an adsorbent, and (d) a set of temperature and humidity sensors as shown in Figure 5. The experiment consisted of several cycles of adsorption and desorption, the same as in the laboratory experiment to distinguish the sample at various stages. The chamber volume is 125,125 mm^3^. With the valve switched to the adsorption mode, the smell of tea leaves was pumped into the sensor chamber from the inlet pipe; when the valve was switched to the desorption mode, the air passed through the adsorbent for cleaning and entered the sensor chamber to wash away the VOCs attached to the sensor’s surface.

During the on-line experiment, first, we put the pipe for the inlet of the tea leaf smell into the tea leaf stack. The VOCs of smell reacted with the sensors, leading to a variation in the sensor’s voltage. After a period, the grassy smell decreased to a lower status, and the tea practitioner shook the tea leaves. We removed the pipe during the shaking. When the practitioners finished the shaking, we inserted the pipe into the stack of tea leaves to monitor the next setting. The standard process of tea manufacturing includes five setting processes: first shaking, second shaking, third shaking, fourth shaking, and fixation. In the end, the result of each experiment showed five periods of that setting process.

### 3.2. Data Analysis

Many methods for the computation of data have been established to distinguish and to classify smell [24]. In this research, we established a novel computation method to analyze the variation of the sensors. After shaking, the grassy smell attained a maximum status; we recorded the voltage value (*V*_F_ or *V*_AS*n*_, *n* = 1, 2, 3, 4, as shown in Figure 3). As the grassy smell decreased to a minimum and balanced level, we recorded the voltage value (*V*_BS*n*_, *n* = 1, 2, 3, 4). For the first shaking monitoring, *V*_BS1_ was divided by *V*_F_ as a relative value for an evaluation of the variation. For the second, third, fourth shaking and fixation monitoring, *V*_BS*n*+1_ was divided by *V*_AS*n*_ as a relative value to evaluate the variation.

(1) $$\mathrm{Relative value}=\frac{V_{BSn+1}}{V_{ASn}},\;n=1,\;2,\;3,\;4$$

In the monitoring experiment, the variation of the E-nose should have complied with the sensory perception of the practitioners. The dominating grassy smell decreased with time during the setting. As the MOS sensor voltage typically decreased when the grassy smell decreased, we assumed that the variation of the sensor decreased during setting.

### 3.3. Result

**Figure 6:**
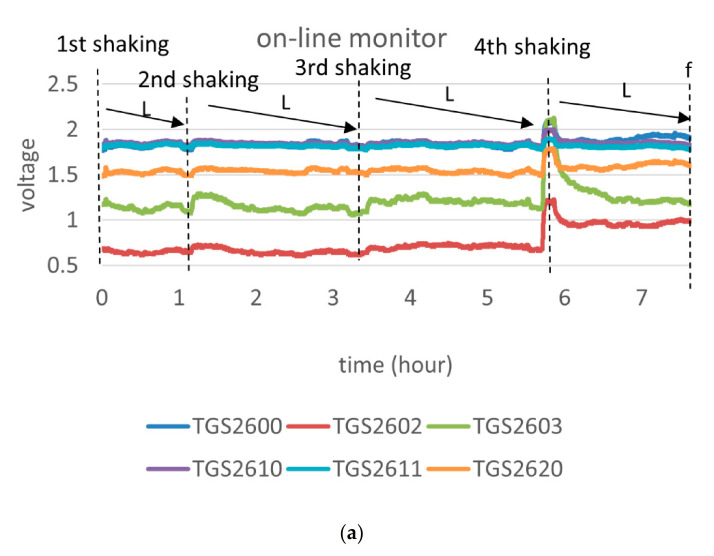

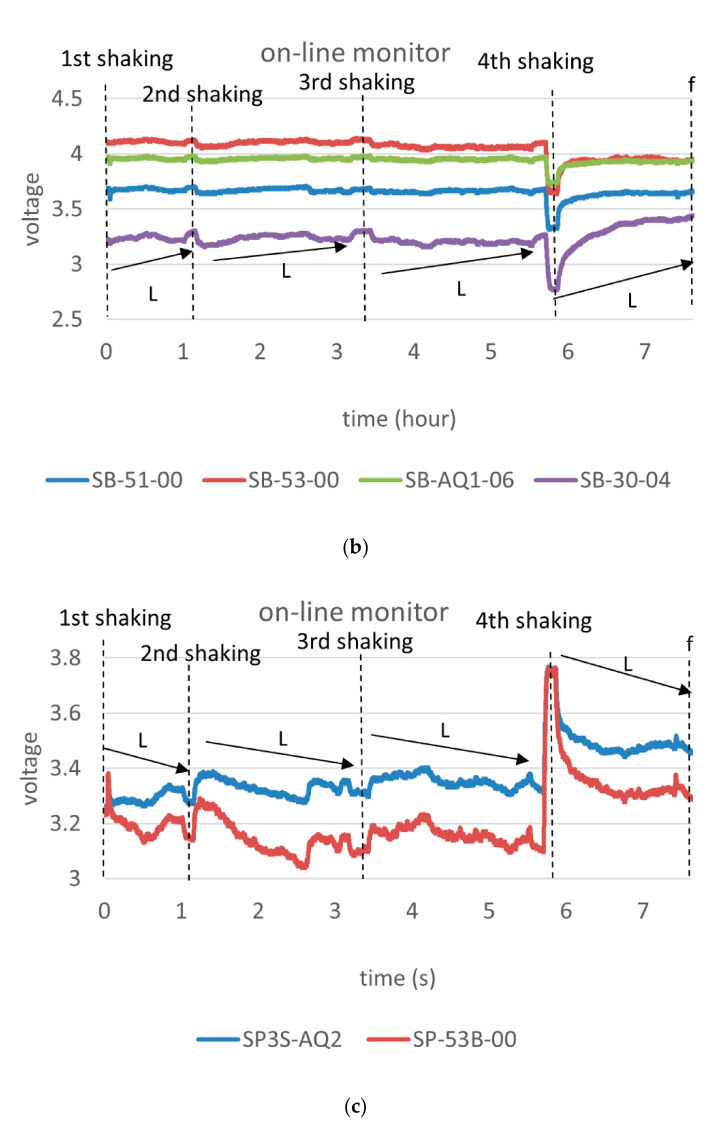
(**a**) Raw data of sensors with prefix ‘TGS-’; (**b**) raw data of sensors with prefix ‘SB-’; (**c**) raw data of sensors with prefix ‘SP-’.

For the on-line experiment, the temperature and humidity were controlled at 35 ± 1 °C and 20 ± 1% RH, respectively. The temperature sensor was installed in the chamber; hence, the heating power from the MOS gas sensor would increase the value of temperature sensor. We only monitored the setting period of the second shaking, third shaking, fourth shaking, and fixation four times. The experiment excluded the first shaking because the effect of the first shaking is to redistribute the moisture from the stalks to the leaves [6,7]. The dashed line in Figure 6 indicates the timing of the four shakings and fixation. Because of an electrical property issue, the measurement voltage of the sensors with prefix ‘SB-’ had a result reversed from that of the sensors with prefix ‘TGS-’ and ‘SP-’. We noticed that the voltage of the TGS and SP series sensors increased at the beginning of the monitoring because the sensors were inserted into the tea sack and exposed to a larger amount of VOC. The SB series sensor showed a contrary variation because of the electrical property issue.

The smell had a dynamic variation during setting, which led to a fluctuation of the voltage during the monitoring. The standard process of tea manufacturing combined two small plates of tea leaves into one large plate after the fourth shaking, which led to a stronger smell and a larger variation of sensors. Practitioners constantly monitored the smell of the tea leaves and compared their olfactory sense to the variation of the E-nose. For standard manufacture, practitioners stopped the setting and shook the tea leaves when the smell of the tea leaves achieved a balance and a low-grass smell. We knew that the values of the sensors also achieved less fluctuation and lower values, which fulfilled our assumption and was equal to our expectation.

The data in Table 2 show that the trend in variations was similar to that of the sensory perception of practitioners. The serial numbers of sensors are demonstrated in green blocks in Table 2. Most of sensors with prefix ‘TGS-’ and ‘SP’ decreased during setting (yellow blocks in Table 2); most sensors with prefix ‘SB-’ showed a contrary variation and increased during setting (yellow blocks in Table 2).

## 4. Conclusions

According to the olfactory sensory perceived by practitioners and previous research, we knew that the samples of tea leaves can be categorized into three groups: before first shaking (BS1), before shaking group, and after shaking group. We applied an E-nose to distinguish and to classify the seven samples; the result was similar to the olfactory sense of the practitioners and previous research. After testing the feasibility of an E-nose on samples with a large difference in the level of grassy smell, we conducted an on-line experiment in a tea factory to monitor the variation of smell during fermentation. The results demonstrated that the variation trends of the E-nose were similar to those of the sensory perception of the tea practitioners, which showed the feasibility of an E-nose in an on-line experiment and elevated the possibility to replace human sensory perception with an E-nose during the manufacture of tea leaves.

## Figures and Tables

**Table 1:** Sensor list (information provided by Figaro and Nissha FIS).

Sensor	Target Gas
TGS-2600	Air contaminants (e.g., hydrogen, carbon monoxide, methane, iso-butane, and ethanol, which exist in cigarette smoke)
TGS-2602	Gaseous air concentrations of odorous gases (e.g., hydrogen, ammonia, ethanol)
TGS-2603	Air contaminants (trimethylamine, methyl mercaptan, etc.)
TGS-2610-C00	Butane, liquefied petroleum gas
TGS-2611-C00	Methane
TGS-2620	Alcohol, vapors of organic solvents
SB-51-00	Hydrogen sulfide
SB-53-00	Ammonia
SB-AQ1-06	Volatile Organic Compounds (VOCs) (for air quality control)
SB-30-04	Alcohol
SP3S-AQ2	VOCs (for air quality control)
SP-53B-00	Ammonia

**Table 2:** Variations in the data after analysis.

Stage	2nd Shaking	3rd Shaking	4th Shaking	Fixation
Process	L	L	L	L
Variation	smaller	smaller	smaller	smaller
TGS2600	1.000→0.989	1.000→0.968	1.000→1.001	1.000→0.912
TGS2602	1.000→1.017	1.000→0.856	1.000→0.957	1.000→0.808
TGS2603	1.000→0.996	1.000→0.838	1.000→0.957	1.000→0.557
TGS2610	1.000→0.995	1.000→0.976	1.000→0.9996	1.000→0.914
TGS2611	1.000→0.995	1.000→0.982	1.000→1.001	1.000→0.946
TGS2620	1.000→0.988	1.000→0.963	1.000→0.999	1.000→0.902
SB-51-00	1.000→1.002	1.000→1.016	1.000→0.999	1.000→1.101
SB-53-00	1.000→0.999	1.000→1.015	1.000→1.004	1.000→1.084
SB-AQ1-06	1.000→1.001	1.000→1.010	1.000→0.9995	1.000→1.056
SB-30-04	1.000→1.000	1.000→1.034	1.000→1.002	1.000→1.242
SP3S-AQ2	1.000→0.998	1.000→0.970	1.000→0.995	1.000→0.919
SP-53B-00	1.000→0.996	1.000→0.932	1.000→0.987	1.000→0.875

## Data Availability

All data were collected and done by the authors.
